# Association of TNFRSF12A Methylation With Prognosis in Hepatocellular Carcinoma With History of Alcohol Consumption

**DOI:** 10.3389/fgene.2019.01299

**Published:** 2020-01-09

**Authors:** Yi Wang, Sina Zhang, Xiaozai Xie, Ziyan Chen, Lijun Wu, Zhengping Yu, Xiaojuan Guo, Gang Chen

**Affiliations:** ^1^ Division of Preventive Medicine, School of Public Health and Management, Wenzhou Medical University, Wenzhou, China; ^2^ Research Center of Evidence-Based Medicine and Clinical Epidemiology, School of Public Health and Management, Wenzhou Medical University, Wenzhou, China; ^3^ School of Public Health, Inner Mongolia Medical University, Hohhot, China; ^4^ Department of Hepatobiliary Surgery, The First Affiliated Hospital of Wenzhou Medical University, Wenzhou, China; ^5^ Division of Clinical Medicine, First School of Clinical Medicine, Wenzhou Medical University, Wenzhou, China

**Keywords:** TNFRSF12A, epigenetics, methylation, hepatocellular carcinoma, prognosis, alcohol abuse

## Abstract

Hepatocellular carcinoma (HCC) is the third leading cause of cancer related death worldwide with a poor prognosis. Alcoholic liver disease accounts for approximately one-third of all HCC cases. Current evidence proved that aberrant over-expression of TNFRSF12A correlates with the severity of disease, making it a likely indicator of disease a more aggressive and worse prognosis outcome. Emerging studies have confirmed that epigenetic changes are critical events in the development and progression of liver cancer. The study to investigate the mechanisms by which alcohol abuse mediated changes in the methylation level of TNFRSF12A affect the occurrence, development and prognosis of HCC were under warranted. Thus, in this study we mined two publicly available datasets to detect the association between DNA methylation level of CpG sites in gene TNFRSF12A and the development of HCC in those with alcohol abuse history. Finally, we discovered that the hypomethylation of two methylation sites—cg00510447 and cg26808293—could identify HCC from other non-HCC liver diseases. Also, hypomethylation of these two sites could identify alcoholic cirrhosis from other non-hepatocellular carcinoma liver diseases. Most important, the prognostic analysis revealed that the hypomethylation of cg00510447 and cg26808293 in HCC patients with alcohol abuse history could predict poor prognosis. Further stratified analyses by gender discovered that in male HCC patients with alcohol abuse history, hypomethylation of cg26808293 signified poor prognosis. The further mechanism analysis revealed that the DNA methyltransferases DNMT3L might regulate TNFRSF12A methylation and affect the occurrence, development and prognosis of HCC, especially in patients with a history of alcohol abuse. These findings provide new insights into the role of epigenetic mechanisms in the transformation of alcoholic liver disease into HCC.

## Introduction

Hepatocellular carcinoma (HCC) is the sixth most common cancer and the third leading cause of cancer deaths in the world (Forner et al., 2012). Alcoholic liver disease is the most common cause of HCC, accounting for about one-third of all HCC cases (Morgan et al., 2004). However, alcohol abuse may not be the only risk factor for HCC. Because even cessation of alcohol consumption, alcoholic liver disease patients still could develop HCC within 1–10 years (Donato et al., 2002). This suggests that alcohol consumption could synergistic with other risk factors for HCC development. Emerging studies have confirmed that epigenetic changes are critical events in the development and progression of liver cancer (Seitz and Stickel, 2007). Epigenetic mechanisms including hypomethylation induced activation of silent oncogenes, loss of imprint, and genomic instability of repetitive DNA sequence. A previous study observed abnormal hypermethylation of specific genes (RASSF1A, GSTP1, CHRNA3, and DOK1) in HCC tumors compared to the cirrhotic liver or normal liver tissue (Lambert et al., 2011). Also, many studies have found that DNA hypomethylation is associated with the development of HCC. Gao et al. reported that hypomethylation of promoter region of LINE-1 gene, especially at the CpG sites 7 and 18, was significantly associated with poor prognosis of liver cancer (Gao et al., 2014). In addition, the interaction between global hypomethylation and regional hypermethylation of genes promotes the progression of liver cancer. For example, DNA hypermethylation induced inactivation of E-cadherin and hypomethylation up-regulated the expression of vimentin, which interact with each other to promote the occurrence and development of liver cancer with poor prognosis (Kitamura et al., 2011).

Tumor necrosis factor alpha (TNFα) is considered to be a critical pro-inflammatory molecule in alcoholic liver injury (El-Serag and Rudolph, 2007). Tumor necrosis factor (TNF) receptor superfamily member 12A (TNFRSF12A; also known as CD266, and TWEAKR) was reported to be upregulated in alcoholic hepatitis (Affo et al., 2013). There are increasing evidence prove that the expression of TNFRSF12A is elevated in various cancers, including breast cancer (Willis et al., 2008), glioma (Tran et al., 2006), esophageal adenocarcinoma (Watts et al., 2007), pancreatic cancer (Han et al., 2002), and HCC (Li et al., 2013; Wang et al., 2017). Moreover, the over expression of TNFRSF12A is associated with poor prognosis in all these tumors. TNFRSF12A is expressed in progenitor cells in response to liver injury (Jakubowski et al., 2005; Tirnitz-Parker et al., 2010). In a mouse model of liver injury maintained on a choline-deficient diet, Bibikova et al. demonstrated that TNFRSF12A promoted the proliferation of liver progenitors (Bibikova et al, 2011). Ethanol administration to these mice resulted in increased expression of TNFRSF12A in the liver, (Tirnitz-Parker et al., 2010). TNFRSF12A is mainly expressed in a subset of hepatocytes and progenitor cells of patients with alcoholic hepatitis. Colocalization of TNFRSF12A with progenitor-derived neonatal hepatocytes in the liver from alcoholic hepatitis patients suggests a potential role for this receptor in progenitor cell differentiation.

In this study, we mined the publicly available database TCGA and GEO aim to: (1) identify the methylation sites in TNFRSF12A which associated with the development of HCC patients with history of alcohol abuse. (2) analyzed the probable regulatory mechanism of TNFRSF12A methylation by methyltransferases in prognosis of HCC with alcoholic hepatitis.

## Materials And Methods

### Data Collection From TCGA And GEO Database

The preprocessed level 3 whole genome methylation microarray data and corresponding clinical data for HCC cases were obtained through the Cancer Genome Atlas (TCGA; https://www.cancer.gov/about-nci/organization/ccg/research/structural-genomics/tcga). At the same time, the mRNA data was extracted from RNA sequencing data on HCC in TCGA. At first, we extracted 371 HCC patient’s data which including 371 cancer samples and 50 paracancerous normal samples from TCGA. After data linking, 345 HCC cases which have completely clinical prognostic information, history of risk factors, mRNA data and methylation data were included in final analysis. The whole genome methylation data of 132 liver disease patients and 34 normal tissues were extracted from the GSE60753 dataset of GPL13534 platform in Gene Expression Omnibus (GEO; https://www.ncbi.nlm.nih.gov/geo). In this study, the criteria to identify the alcohol abuse were as same as the criteria identified in the GSE60753 dataset (Hlady et al., 2014). DNA methyltransferases (DNMT1, DNMT2, DNMT3A, DNMT3B, DNMT3L) and TNFRSF12A gene mRNA data were extracted from DNA microarray in 15 patients with alcoholic hepatitis from the GSE28619 dataset of GPL570 platform in GEO.

### Methylation and Gene Expression Analysis

Gene expression was defined using the raw read count and log2 transformed normalized count. Whole genome methylation data were measured by Illumina Human methylated 450k microarrays for HCC in TCGA dataset. In GSE60753 dataset, the methylation level was measured by Illumina Human methylated 27k microarrays. The methylation level of CpG islands were represented as β values. B = Intensity of the methylated allele (M)/[Intensity of the unmethylated allele (U) + Intensity of the methylated allele (M) + 100] (Jaenisch et al., 2003). The β values will map to genome (methylation site/gene) and used to perform DNA methylation analysis. Any methylation site corresponding to the TNFRSF12A gene without a β value was excluded. The final identified sites list included 19 methylation sites identified from the TCGA database, and 12 methylation sites identified from the GSE60753 dataset (**Table 1**). We used the median value of the methylation level as the cutoff point to divide the patients into hypomethylation and hypermethylation groups. Similarly, the median value of TNFRSF12A expression was used as the cutoff point to divide patients into high expression group and low expression group.

**Table 1:** Methylation sites corresponding to TNFRSF12A in TCGA database and GSE60753 dataset.

**Methylation: sites**	**Is there a β value? (yes or no)**		**Start**	**End**	**Gene symbol**	**Feature type**
	**TCGA**	**GSE60753**				
**cg00510447**	Yes	Yes	3021607	3021608	TNFRSF12A	S_Shore
**cg26808293**	Yes	Yes	3022206	3022207	TNFRSF12A	S_Shore
**cg06209210**	Yes	Yes	3020915	3020916	CLDN6;TNFRSF12A	Island
**cg15460516**	Yes	Yes	3020248	3020249	CLDN6;TNFRSF12A	Island
**cg05336707**	Yes	Yes	3020469	3020470	CLDN6;TNFRSF12A	Island
**cg20195987**	Yes	Yes	3021145	3021146	CLDN6;TNFRSF12A	Island
**cg08798492**	Yes	Yes	3020268	3020269	CLDN6;TNFRSF12A	Island
**cg06097320**	Yes	Yes	3020244	3020245	CLDN6;TNFRSF12A	Island
**cg02105042**	Yes	Yes	3019455	3019456	CLDN6;TNFRSF12A	N_Shore
**cg02199397**	Yes	No	3018314	3018315	CLDN6;TNFRSF12A	Island
**cg08934846**	Yes	No	3018344	3018345	CLDN6;TNFRSF12A	Island
**cg07384961**	Yes	No	3018127	3018128	CLDN6;TNFRSF12A	Island
**cg10293804**	Yes	No	3018528	3018529	CLDN6;TNFRSF12A	S_Shore
**cg08563839**	Yes	No	3017499	3017500	CLDN6;TNFRSF12A	N_Shore
**cg06036912**	Yes	No	3018339	3018340	CLDN6;TNFRSF12A	Island
**cg17865114**	Yes	No	3017758	3017759	CLDN6;TNFRSF12A	Island
**cg06080729**	Yes	No	3017625	3017626	CLDN6;TNFRSF12A	Island
**cg08935125**	Yes	No	3018084	3018085	CLDN6;TNFRSF12A	Island
**cg02732252**	Yes	No	3018013	3018014	CLDN6;TNFRSF12A	Island
**cg00572487**	No	Yes	3020062	3020063	CLDN6;TNFRSF12A	N_Shore
**cg06712763**	No	Yes	3020056	3020057	CLDN6;TNFRSF12A	N_Shore
**cg16657615**	No	Yes	3020629	3020630	CLDN6;TNFRSF12A	Island
**cg06473109**	No	No	3018466	3018467	CLDN6;TNFRSF12A	S_Shore
**cg10298701**	No	No	3018474	3018475	CLDN6;TNFRSF12A	S_Shore
**cg14618624**	No	No	3018381	3018382	CLDN6;TNFRSF12A	S_Shore
**cg20035459**	No	No	3018706	3018707	CLDN6;TNFRSF12A	S_Shore

### Statistical Analysis

The continuous variables were expressed as mean ± standard deviation or median (quartile range), and categorical variables were presented as frequencies (percentages). Wilcoxon rank sum test was used to compare the differences of methylation level between normal and HCC tissues, or between normal and liver disease tissues (*p*-value <1.0E−7 were considered as statistically significant). Independent samples t-test was used to analyze the expression differences of TNFRSF12A gene between HCC patients with alcohol abuse risk factor and non-alcoholic risk factor. Kruskal–Wallis H test was used to compare multiple sets of independent samples. Cox proportional hazard regression model was conducted to analyze the effect of methylation level of each site of TNFRSF12A identified in the TCGA database on the overall survival rate of HCC patients when adjusted for covariables. Kaplan–Meier survival curve analysis and log-rank method were used to compare the survival time of different levels of study factors. A linear correlation model was performed to evaluate the relationships between the variables and using Pearson correlation coefficient or Spearman rank correlation coefficient to present the result. Unless otherwise indicated, all statistical tests were two-sided and *p*-value < 0.05 were considered as statistically significant. All analyses were performed using R (V 3.4.2) software. Normalization pretreatment of methylation data was done using the R *limma* package; Kaplan–Meier survival analysis and Cox proportional hazard regression analysis were performed by R *survival* package. The interaction analysis among the methylation sites, clinicopathological features, and the survival status were conduct by R *HH* package ancova function. Linear correlation analysis between variables was conducted by R *Hmisc* package and the visualization of the correlation coefficient between DNA methylase level, TNFRSF12A gene expression level and TNFRSF12A gene methylation site were performed by R *PerformanceAnalytics* package chart.correlation function.

## Results

### Clinicopathological Characteristics of Patients In TCGA And GEO Database

Among the 345 HCC patients from TCGA, 67.8% was male, and 32.2% was female, with an average age of 59.34 ± 13.13 years; 144 (41.7%) were Asians, 192 (55.7%) were non-Asian, and nine of unknown race. These HCC patients included 116 cases that had a history of alcohol abuse, 140 cases that had other non-alcoholic risk factors, and 89 cases that had no major risk factors. The median survival time of these patients was 18.92 months. The main demographic and clinicopathological characteristics of HCC patients from TCGA were presented in **Table 2**. Among the woabuse as risk factor (HCC-EtOH); three were cryptogenic cirrhosis; two were biliary cirrhosis; four were genetic cirrhosis; 2 were immune cirrhosis; 21 cases were cirrhosis with chronic alcohol abuse(cirr-EtOH); six cases were cirrhosis with infection of HBV(cirr-HBV); 39 cases were cirrhosis with infection of hepatitis C virus(cirr-HCV); five cases of cryptogenic HCC; 12 cases were HCC with HCV(HCC-HCV); 21 cases of unclassified liver and two cases of biliary tumor. There was no demographic information of patients in GSE60753 dataset.

**Table 2:** Clinicopathological characteristics of the HCC patients in TCGA database.

Clinicopathological variables	TCGA (n = 345)	Percentage (%)
**Gender**		
Male	234	67.80
Female	111	32.20
**Race**		
Asians	144	41.70
Other	192	55.70
Unknown	9	2.60
**Residual tumor status**		
With tumor	140	40.60
Tumor-free	190	55.10
Unknown	15	4.40
**Recurrence**		
YES	161	46.70
NO	184	53.30
**BMI**		
< 25	186	53.90
≥25	159	46.10
**HBV**		
Positive	102	29.56
Negative	232	67.25
Unknow	11	3.19
**HCV**		
Positive	52	15.07
Negative	282	81.74
Unknow	11	3.19
**Risk factor history**		
Alcohol abuse^*^	116	33.60
Other risk factor without alcoholic abuse	140	40.60
No history of primary risk factors	89	25.80
**Histologic Grade**		
Grade 1–Grade 2	217	62.90
Grade 3–Grade 4	125	26.20
Unknown	3	0.90
**TNM stage^¶^**		
Stage 1	174	50.40
Stage 2	80	23.20
Stage 3	91	26.40

^*^Among the 116 patients with alcohol abuse history, 24 patients were HBV positive and 19 patients were HCV positive. **^¶^**Stage 1 includes stage I; Stage 2 includes stage II; stage 3 includes stages IIIA, IIIB, IIIC, IV (IVA, IVB).

### Methylation Status in the TNFRSF12A Gene

**Figure 1:**
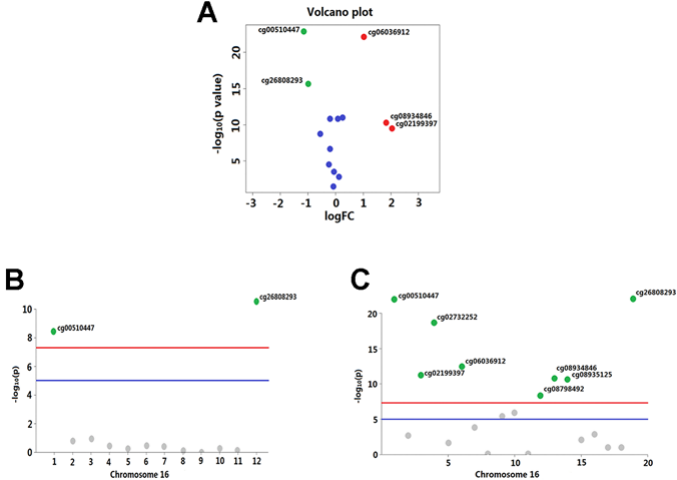
Significant differences in methylation levels at each site of TNFRSF12A gene in liver diseases. **(A)** A volcano plot depicting methylated level of each sites of TNFRSF12A between HCC tumor and non-tumor tissues from TCGA. X-axis is represented as log10 fold change and Y-axis as −log10 of *p*-value. Methylation with logFC > 0.8 and *p < *1.0E−7(marked as red plots) or logFC <−0.8 and *p <* 1.0E−7(marked as green plots) were considered differentially significant. **(B)** A Manhattan plot showing the methylation level of 12 sites identified in the GSE60753 dataset. Methylation of cg00510447 and cg26808293 in TNFRSF12A was significantly different between normal and liver disease tissues. (blue line: *p* = 1.0E−5; red line: *p* = 1.0E−7) **(C)** Manhattan plot showing methylation level of the 19 sites in TNFRSF12A from the TCGA (blue line: *p* = 1.0E−5; red line: *p* = 1.0E−7).

**Figure 2:**
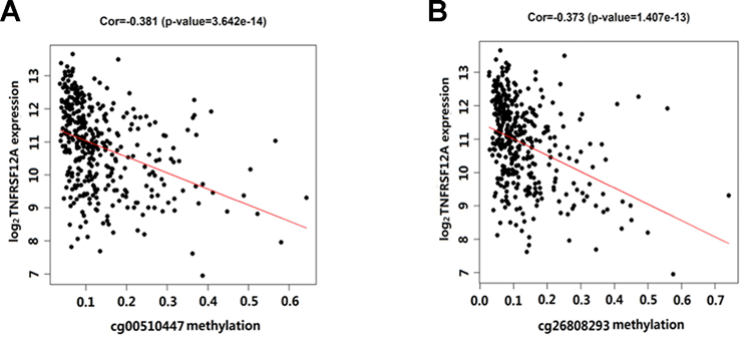
Scatterplot showing the correlation between methylation levels of cg00510447 and cg26808293 and TNFRSF12A expression in HCC cases. **(A)** The correlation between cg00510447 methylation level and TNFRSF12A expression level. **(B)** The correlation between cg26808293 methylation level and TNFRSF12A expression level.

Overall, out of the 19 methylation sites corresponding to TNFRSF12A identified in the TCGA, significant differences were seen in five methylation sites between HCC cancerous and paracancerous tissues (*p* <1.0E−7, |logFC| >0.8). Among these five sites, cg00510447 and cg26808293 had a logFC<−0.8; cg06036912, cg08934846, and cg02199397 had a logFC >0.8 (**Figure 1A**). Out of the 12 sites identified in the GSE60753 dataset, only methylation of cg00510447 and cg26808293 were significantly different between normal and liver diseases tissues (**Figure 1B**). Moreover, the methylation level of cg00510447 and cg26808293 are the highest in the 19 methylation sites identified in TCGA between HCC and cancer adjacent tissues (**Figure 1C**). Linear correlation analysis demonstrated a statistically significant correlation between three methylation sites (cg00510447, cg26808293, cg02105042) and the expression of TNFRSF12A. Among them, the methylation levels of cg00510447 and cg26808293 have strongest negative correlation with the expression level of TNFRSF12A gene (*r* = −0.381 and *r* = −0.373, respectively). (**Table 3**, **Figure 2**).

**Table 3:** The correlation analysis between methylation levels of methylation sites and expression level of TNFRSF12A.

Methylation sites	Correlation coefficient(*r*)	*p* value
**cg00510447**	−0.381	3.642E−14
**cg26808293**	−0.373	1.407E−13
**cg02105042**	−0.142	0.0064
**cg08563839**	0.086	0.0998
**cg06209210**	−0.017	0.7437
**cg15460516**	−0.001	0.9821
**cg20195987**	−0.063	0.2306
**cg08935125**	−0.050	0.3386
**cg05336707**	0.041	0.4313
**cg02199397**	−0.041	0.4276
**cg06080729**	0.083	0.1132
**cg08934846**	0.016	0.7525
**cg07384961**	−0.008	0.8710
**cg06097320**	−0.052	0.3231
**cg08798492**	0.041	0.4352
**cg10293804**	−0.003	0.9507
**cg17865114**	0.056	0.2803
**cg02732252**	−0.023	0.6669
**cg06036912**	−0.049	0.3486

### Predictive Value Of Methylation Sites in the TNFRSF12A Gene for HCC Prognosis

The Cox proportional hazards regression model was used to analyze the relationship between the methylation level of TNFRSF12A and survival of HCC patients which adjusting for age, gender, race, histological grade, residual tumor status(presence or absence), BMI, recurrence status, history of HCC risk factors, and TNM staging. **Table 4** shows the prognostic analysis results of the 19 methylation sites ranked by the *p*-values. The prognostic analysis results showed that hypermethylation of cg00510447 (HR = 0.03, *p* = 0.04) and cg26808293(HR = 0.02, *p* = 0.02) may offer a favorable prognosis of HCC. Hypermethylation of cg15460516 (HR = 3.93E13, *p* = 0.001) and cg06209210 (HR = 8.49E6, *p* = 0.012) was identified as a risk factor affecting the prognosis of HCC.

**Table 4:** Cox proportional hazard regression analysis of the relationship between the methylation sites of TNFRSF12A and survival rate of HCC patients from TCGA.

Methylation sites	HR (95%CI)^¶^	*p* value
**cg15460516**	3.93E+13(7.47E+5–2.07E+21)	0.001
**cg26808293**	0.02(0–0.24)	0.002
**cg00510447**	0.03(0–0.31)	0.004
**cg06209210**	8.49E+6(34.83–2.07E+12)	0.012
**cg06080729**	9.28(0.63–136.21)	0.104
**cg05336707**	1.44E+17(0–1.10E+43)	0.194
**cg08935125**	0.41(0.09–1.77)	0.231
**cg08934846**	1.58(0.74–3.37)	0.235
**cg20195987**	0(0–1715421.33)	0.265
**cg02199397**	1.67(0.58–4.80)	0.339
**cg08798492**	10.34(0.05–2009.14)	0.385
**cg07384961**	0.67(0.26–1.76)	0.419
**cg10293804**	1.30(0.60–2.83)	0.510
**cg06097320**	0(0–16665008.15)	0.592
**cg17865114**	1.80(0.14–23.41)	0.653
**cg02732252**	1.25(0.41–3.82)	0.699
**cg02105042**	0.89(0.26–3.05)	0.853
**cg08563839**	1.23(0.10–14.47)	0.868
**cg06036912**	1.05(0.40–2.79)	0.916

^¶^The model adjusted for age, gender, race, histological grade, residual tumor status, BMI, recurrence status, history of HCC risk factors, and TNM stage.

### Stratified Survival Analysis of cg00510447 and cg26808293 in HCC Patients With and Without Alcohol Abuse

**Figure 3:**
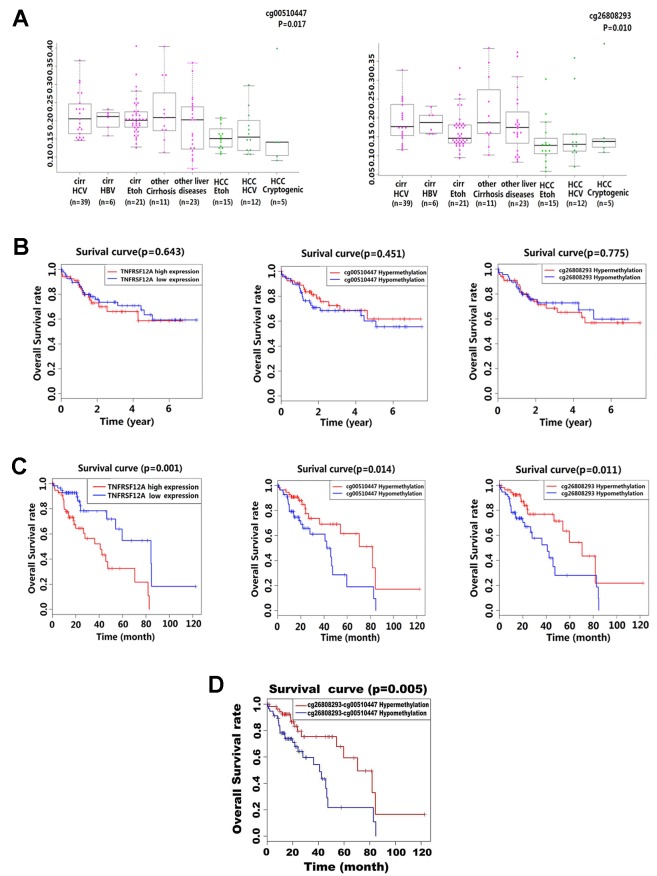
Methylation levels of cg00510447 and cg26808293 in liver disease and prognostic association in HCC patients with or without alcoholic risk factors. **(A)** Beeswarm plot showing significant difference in the methylation levels of the two methylation sites in 132 cases of multiple liver disease cases (15 cases of HCCEtoh; 21 cases of cirrEtoh; six cases of cirrHBV; 39 cases of cirrHCV; five cases of Cryptogenic HCC; 12 cases of HCC HCV; 11 cases of other cirrhosis; 23 cases of other liver disease), cg00510447 and cg26808293, which significantly indicated p value < 0.05. **(B)** Kaplan–Meier survival curves of high and low expression of TNFRSF12A and hypermethylation and hypomethylation of cg00510447 and cg26808293 in 140 HCC cases with non-alcoholic risk factors. **(C)** Kaplan–Meier survival curves of high and low expression of TNFRSF12A and hypermethylation and hypomethylation of cg00510447 and cg26808293 in 116 cases of HCC with a risk alcohol consumption. **(D)** Kaplan–Meier survival curve of the association between hypomethylation of cg26808293 combined with hypomethylation of cg00510447 and hypermethylation of cg26808293 combined with hypermethylation of cg00510447in hepatocellular carcinoma cases with risk factors of alcohol consumption.

Kruskal–Wallis analysis of the methylation level in 132 liver disease cases from GSE60753 dataset showed that cg00510447 and cg26808293 were hypomethylated in HCC cases compared to other liver diseases (*p* = 0.017 and *p* = 0.010, respectively). Further analysis showed that in those non-HCC liver disease cases, the methylation level of these two sites was lower in alcoholic cirrhosis than other liver diseases (**Figure 3A**). Then, the stratified survival analysis show that in 140 HCC cases from TCGA which without alcoholic abuse risk factor, there was no significant difference of prognostic value between different level of TNFRSF12A expression and different methylation level of cg00510447 and cg26808293 (**Figure 3B**). However, in 116 HCC cases which have the alcohol abuse risk factor, there was a significant prognostic association of TNFRSF12A high expression, hypomethylated cg00510447 and hypomethylated cg26808293 (**Figure 3C**). Furthermore, hypomethylation of cg00510447 combined with hypomethylation of cg26808293 predicted worse prognosis in HCC patients with alcohol abuse risk factor (**Figure 3D**).

### Interaction Between Methylation Sites and Clinicopathological Characteristics

**Figure 4:**
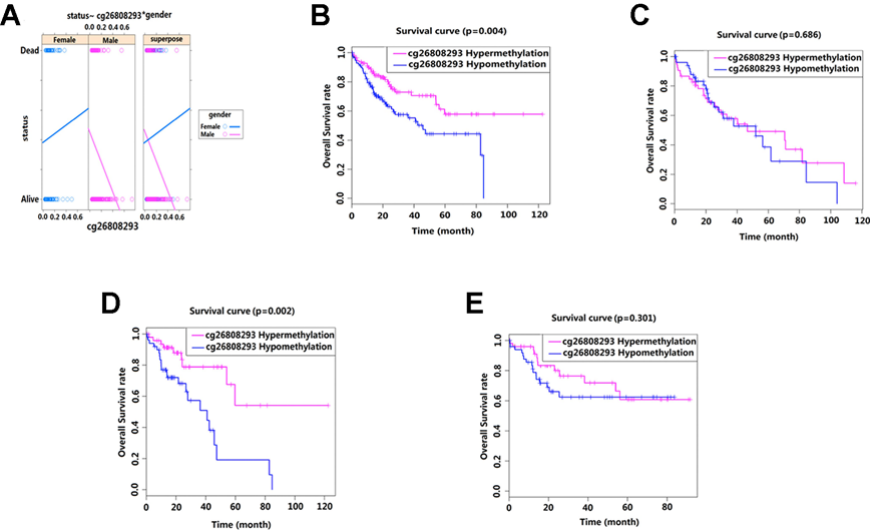
Interactions between methylation site cg26808293 and clinicopathological variables. **(A)** The methylation level of cg26808293 interacts with the gender variable in the survival state. The methylation level of cg26808293 was used as a continuous variable, the gender of HCC was used as an attribute variable, and the patient’s prognosis was the response variable. **(B)** The Kaplan–Meier survival curve of hypermethylation and hypomethylation of cg26808293 in male HCC cases. **(C)** The Kaplan–Meier survival curve of hypermethylation and hypomethylation of cg26808293 in female HCC cases. **(D)** The Kaplan–Meier survival curve of hypermethylation and hypomethylation of cg26808293 in male HCC cases with alcohol abuse history. **(E)** The Kaplan–Meier survival curve of hypermethylation and hypomethylation of cg26808293 in male HCC cases without alcohol abuse history.

We conducted interaction analysis using methylation levels of cg00510447 and cg26808293 as continuous variables, the clinicopathological characteristics including age, gender, race, histological grade, presence or absence of residual tumors, BMI, recurrence, hepatocellular carcinoma risk factor history, TNM stage as attribute covariables, and overall survival status of HCC as the response variable. Results showed that only the interaction between methylation level of cg26808293 and gender was statistically significant (*p* = 0.036) (**Table 5**, **Figure 4A**). Therefore, we conducted further stratified survival analysis by different gender. Results showed that the prognostic predictive value of cg26808293 in males was better than females. Kaplan–Meier survival curve showed that the prognosis of hypomethylation in cg26808293 worse than hypermethylation for male HCC patients (*p* = 0.004) (**Figures 4B**, **C**). Moreover, the stratified analysis for male HCC patients with or without history of alcohol abuse showed that the prognosis of hypomethylated cg26808293 was significantly lower in male HCC patients with a history of alcohol abuse (*p* = 0.002). However, these differences have not observed in those male HCC patients with non-alcoholic risk factors (**Figures 4D, E**).

**Table 5:** Interaction of two methylation sites (cg00510447 and cg26808293) with clinicopathological characteristics.

Response: survival status					
Variables	*p* value	Variables	*p* value	Variables	*p* value
**cg00510447**	0.0225	**cg00510447**	0.0241	**cg00510447**	0.0223
**race**	0.0151	**BMI**	0.8462	**risk factors**	0.0058
cg00510447*race	0.2970	cg00510447*BMI	0.3733	cg00510447*risk factors	0.4668
**cg26808293**	0.0091	**cg26808293**	0.0096	**cg26808293**	0.0090
**race**	0.0183	**BMI**	0.8974	**risk factors**	0.0065
cg26808293*race	0.5410	cg26808293*BMI	0.0995	cg26808293*risk factors	0.9900
**cg00510447**	0.0235	**cg00510447**	0.0202	**cg00510447**	0.0192
**age**	0.4150	**recurrence**	<0.0001	**stage**	<0.0001
cg00510447*age	0.0667	cg00510447*recurrence	0.1576	cg00510447*stage	0.0707
**cg26808293**	0.0097	**cg26808293**	0.0079	**cg26808293**	0.0076
**age**	0.4461	**recurrence**	<0.0001	**stage**	<0.0001
cg26808293*age	0.2320	cg26808293*recurrence	0.7810	cg26808293*stage	0.7015
**cg00510447**	0.0230	**cg00510447**	0.0167	**cg00510447**	0.0236
**gender**	0.0416	**residual tumor**	<0.0001	**grade**	0.0704
cg00510447*gender	0.1142	cg00510447*residual tumor	0.6943	cg00510447*race	0.9983
**cg26808293**	0.0091	**cg26808293**	0.0058	**cg26808293**	0.0093
**gender**	0.0649	**residual tumor**	<0.0001	**grade**	0.0652
cg26808293*gender	0.0363	cg26808293*residual tumor	0.1111	cg26808293*grade	0.4151

### Correlation Between Methylation Sites in TNFRSF12A and DNA Methylase in HCC

**Figure 5:**
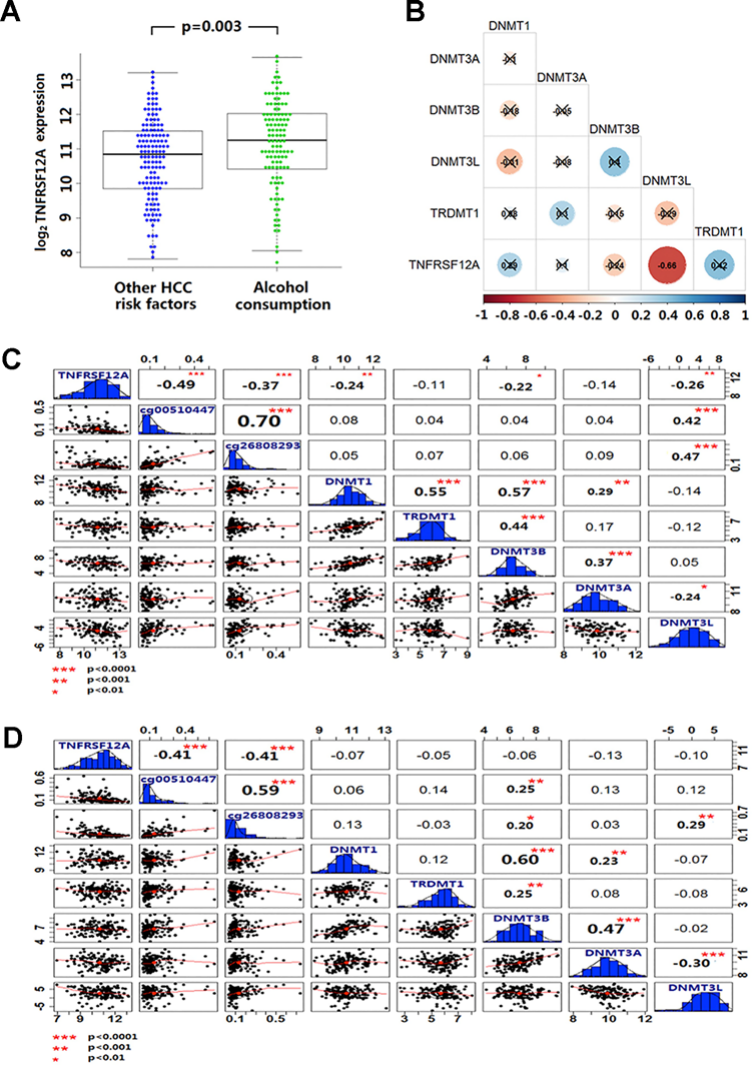
Alcohol and non-alcoholic consumption on DNA methyltransferase expression in liver disease and the correlation between cg00510447 and cg26808293 methylation and TNFRSF12A expression. **(A)** Beeswarm plot showing differences in expression of TNFRSF12A in HCC cases with alcohol abuse history and without alcohol abuse history. **(B)** Correlation coefficient plot shows the association of TNFRSF12A with expression of the five DNA methylases in 15 cases of alcoholic hepatitis. **(C)** Correlation coefficient plot shows the expression level of TNFRSF12A and the methylation level of cg00510447 and cg26808293 and the correlation of expression of five DNA methyltransferases in 116 cases of hepatocellular carcinoma with alcohol consumption. **(D)** Correlation coefficient plot showing the expression level of TNFRSF12A and the methylation level of cg00510447 and cg26808293 and correlation with the five DNA methylases in 140 cases of hepatocellular carcinoma without alcohol abuse.

Expression of TNFRSF12A was higher in HCC cases with a history of alcohol abuse (*p* = 0.003) (**Figure 5A**). In the GSE28619 dataset, out of five DNA methylases, only DNMT3Lwas identified to be negatively correlated with TNFRSF12A expression (*r* = −0.66, *p <* 0.001) in 15 cases of alcoholic hepatitis (**Figure 5B**). Expression of DNMT1, DNMT3B and DNMT3L was identified to be negatively correlated with the expression of TNFRSF12A in 116 HCC with alcohol abuse (*r* <−0.22, *p <* 0.01). Moreover, only the expression of DNMT3L was positively correlated with the methylation level of cg00510447 and cg26808293 (*r >*0.40, *p <* 0.001) (**Figure 5C**).Compared to alcoholic HCC patients, there was no DNA methylases to be identified have correlation with the expression of TNFRSF12A and DNMT3L expression was not correlated to cg00510447 methylation level in non-alcoholic HCC patients (*p >* 0.05). Even though there was positive correlation between DNMT3L expression and the methylation level of cg26808293, the correlation trend was weak compare to those in alcoholic HCC patients (*r* = 0.29, *p <* 0.001) (**Figure 5D**).

## Discussion

In the present study, we analyzed the methylation level of TNFRSF12A in HCC and other liver diseases through data mining from publicly available databases. Firstly, we found a higher expression of TNFRSF12A have worse prognosis in HCC patients with a history of alcohol abuse. Further analysis revealed both the cg00510447 and cg26808293 sites in the TNFRSF12A gene were significantly hypomethylated in HCC patients than normal tissues, similarly, these were observed in cases of alcoholic hepatitis. In cases of HCC with a history of alcohol abuse, these two sites hypomethylation predict a worse prognosis than hypermethylation. Furthermore, cg00510447 as well as cg26808293, was negatively correlated with TNFRSF12A expression. Related studies have reported that in the process of tumorigenesis, accompanied by hypermethylation of CpG, the methylation of CpG sites with gene scatter can often occur because of insufficient methylation of the entire genome (Ehrlich, 2002). Hypomethylation may be associated with abnormal activation of individual genes, which will cause extensive changes in gene expression patterns and is the molecular basis for genomic instability. DNA methylation is the central epigenetic mechanism of human gene expression regulation, and studies have shown that this mechanism change is one of the major molecular variants of malignant tumors (Jones and Takai, 2001; Esteller, 2008).

In this study, we conducted the survival analysis combined the cg00510447 and cg26808293 methylation levels in HCC patients with a history of alcohol abuse. The result shows that combination of these two CpG sites hypomethylation were predictors of poor prognosis in HCC patients with a history of alcohol abuse than hypermethylation of these two sites. These results suggested that hypomethylation of cg00510447 and cg26808293 may activated the expression of TNFRSF12A, resulting in poor prognosis in HCC patients. We also found that hypomethylated cg00510447 and cg26808293 can distinguish alcoholic cirrhosis from other non-HCC liver diseases. Hence, it is safe to imply that the hypomethylation of these two points may provide a new mechanism by which alcoholic hepatitis progresses to HCC. Except for gender, none of the clinicopathological variables showed any interaction between methylation of cg00510447 and survival of patients. In this study, hypomethylation of cg26808293 predicted a poor prognosis only in males with HCC who had a history of alcohol consumption. It is probably because males are likely to consume alcoholic beverages more than women (Greenfield et al., 2010). There is a risk factor for alcohol consumption in long-term natural selection. Hypomethylation of cg26808293 was a predictor of poor prognosis whereas hypermethylation could predict a better prognosis in HCC patients with a history of alcohol consumption. Hypermethylation of cg26808293 could protect males with alcoholic HCC by improving disability-adjusted life year and reducing the burden of disease.

DNA methyltransferases (DNMTs) catalyze the transfer of methyl groups from S-adenosylmethionine to DNA. The mammalian DNMT family has five members-DNMT1, DNMT2, DNMT3A, DNMT3B, and DNMT3L, out of which DNMT1 is the most critical enzyme that regulates methylation of newly synthesized DNA strands under the guidance of a methylated template. Even a transient inhibition of DNMT1 can cause long-term stable demethylation (Loriot et al., 2006). While targeted cleavage of either DNMT1 or DNMT3b has little effect on DNA methylation and growth in colon cancer HCT116 cells, simultaneous inactivation of both the methyltransferases results in hypomethylation of a wide range of genomes (Rhee et al., 2002).In this study, we analyzed the correlation between five DNA methyltransferases (DNMT1, DNMT2, DNMT3A, DNMT3B and DNMT3L) and TNFRSF12A expression data from 15 alcoholic hepatitis patients in the GSE28619 dataset. Only a significant negative correlation was found between DNMT3 and TNFRSF12A expression levels. We further analyzed the relationship among the expression levels of the five DNA methyltransferases, TNFRSF12A expression and methylation levels of cg00510447 and cg26808293 in 116 HCC patients with a history of alcohol consumption. Results showed a significant negative correlation between DNMT1, DNMT3b and DNMT3L and TNFRSF12A expression, and a significant positive relationship with the methylation of cg00510447 and cg26808293. We found that there was no linear correlation between these factors in HCC patients with no history of alcohol consumption. Also, there was a significant but weak linear correlation with the methylation level of cg26808293. Based on the above findings, we speculate that alcohol inhibits DNA methyltransferases, specifically, DNMT1, DNMT3B, DNMT3L, through a metabolic pathway, causing a decrease in 5mC content and alteration in methylation characteristics. In the process of carcinogenesis, alcohol causes expansion of cancerous cell clones. Due to the limitation of maintaining the capacity of DNMT3L during DNA replication, the degree of methylation of the corresponding methylation site is decreased, increasing the expression of TNFRSF12A, thereby affecting the survival prognosis of HCC patients with a history of alcohol consumption. The existence of such epigenetic mechanisms remains to be verified by future studies.

We also performed a Spearman correlation analysis of all mRNA expression levels and TNFRSF12A methylation levels (cg00510447 and cg26808293 two-point averaging levels) in the TCGA-hepatocarcinoma program, the correlation coefficient |*r*| > 0.3 is included in the KEGG pathway. The results of the analysis showed that mRNA associated with TNFRSF12A methylation was mainly enriched into five KEGG pathways, including Carbon metabolism. (**Supplementary Figure S1**). Searching for DNMT3L in protein and protein interaction search tools (String) found that eight related proteins are enriched in the protein interaction network and related to KEGG pathway-Alcoholism (**Supplementary Figure S2**). In conclusion, whether alcohol affects DNA methylase through one-carbon metabolism pathway and thus reduces the degree of methylation of TNFRSF12A through epigenetic effects, ultimately affecting the prognosis of patients with HCC hepatocellular carcinoma remains to be determined.

Despite a lack of experimental studies to determine the causality of functional and regulatory pathways, our study provided a novel concept that epigenetic mechanisms are involved in the development of HCC, and eventual transformation of alcoholic liver disease into HCC. Our results showed that the methylation status of cg00510447 and cg26808293 corresponding to TNFRSF12A was negatively correlated to the prognosis of HCC with alcoholic cirrhosis. These findings suggested that quantitative detection of methylation at these two points can serve as predictive markers of prognosis in HCC, especially in those with alcohol abuse history. However, mechanisms underlying the hypomethylation of TNFRSF12A in HCC with alcohol abuse history remain unclear. Some studies have shown that this may be related to the dysfunction of methyltransferases. Most significantly, the highly dynamic nature of epigenetic mechanisms offers hope for the discovery of novel therapies in liver disease. Objectively, changing the methylation status of genes through medications or gene therapy can influence the development of liver diseases. DNA methylation can be used as a marker for an early stage of hepatocellular carcinoma. Our study suggested that drugs can be developed to intervene early in the aberrant methylation of TNFRSF12A, transcriptional regulation of the TNFRSF12A gene to improve the survival of patients with HCC with a history of alcohol consumption.

## Data Availability Statement

All datasets generated for this study are included in the article/**Supplementary Material**.

## Author Contributions

YW, SZ, XX, ZC, and LW conducted statistical analyses of the whole data. YW, SZ, and GC wrote the draft and revised manuscript. YW, SZ, and XG provided statistical expertise and were involved in data analysis and interpretation of results. GC and ZY conceived and supervised the study. All co-authors reviewed and made contributions to the final manuscript.

## Funding

This work was funded by the National Natural Science Foundation of China (Grant No. 81201953, 81772628 to GC and 81703310 to YW); the Natural Science Foundation of Zhejiang Province (No.LY17H160047 to GC); the Public Projects of Zhejiang Province (No. 2018C37114 to YW, No. 2016C37127 to ZY).

## Conflict of Interest

The authors declare that the research was conducted in the absence of any commercial or financial relationships that could be construed as a potential conflict of interest.

## Supplementary Material

Supplementary Figure S1 KEGG pathway enrichment analysis of all mRNA expression and TNFRSF12A methylation in HCC in the TCGA Database.

Supplementary Figure S2 DNMT3L and Alcoholism KEGG pathway related protein interaction network in String database.
