# Supplementary figures and images for: Association of TNFRSF12A Methylation With Prognosis in Hepatocellular Carcinoma With History of Alcohol Consumption
Source: Front Genet. 2020 Jan 9;10:1299. doi: 10.3389/fgene.2019.01299 (PMC6964049; doi:10.3389/fgene.2019.01299)

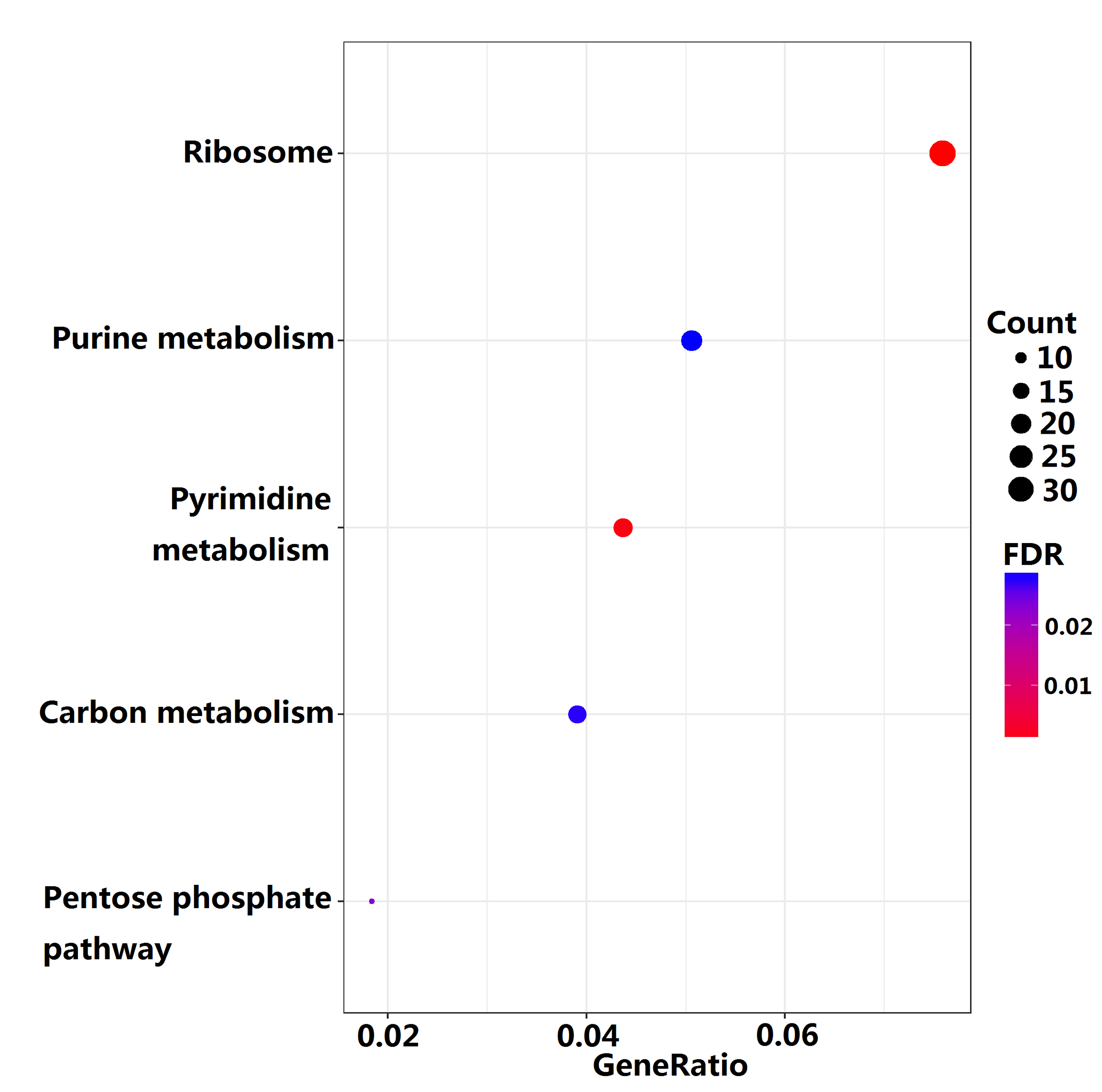

Supplement: Supplementary Figure S1 — KEGG pathway enrichment analysis of all mRNA expression and TNFRSF12A methylation in HCC in the TCGA Database. [file Image_1.tif]

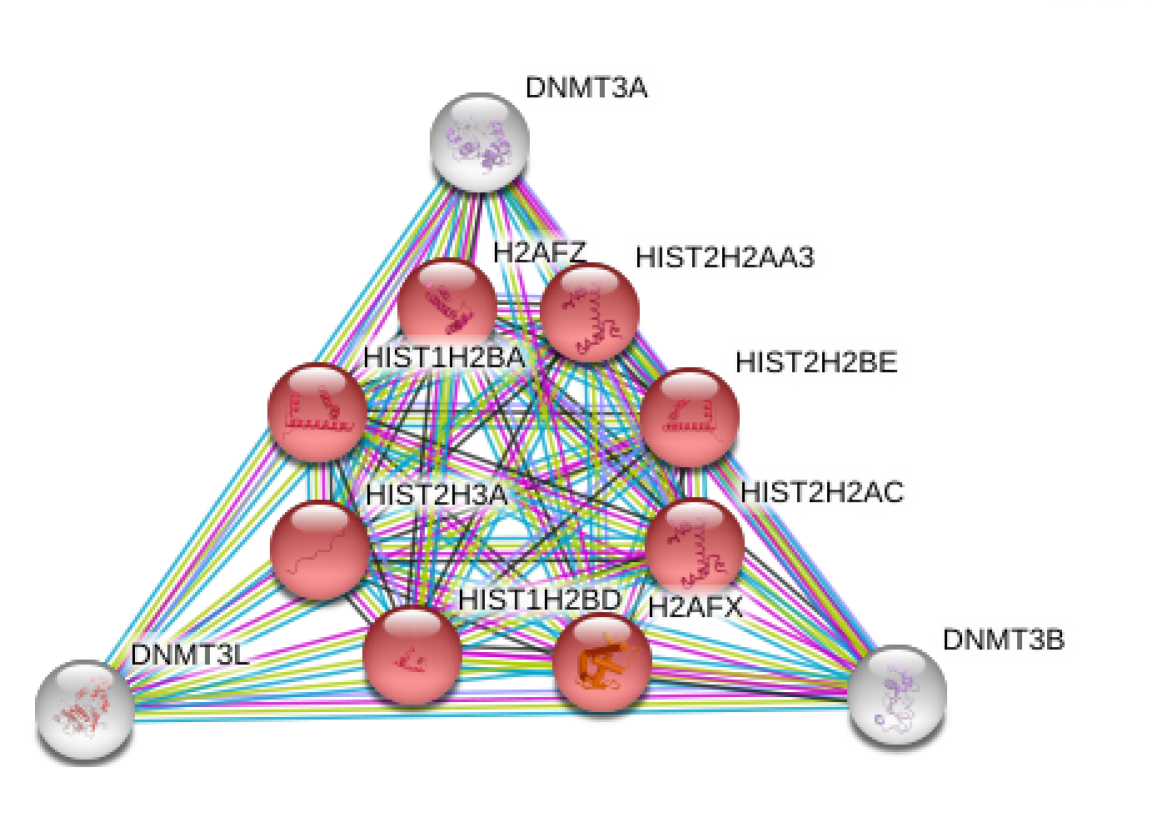

Supplement: Supplementary Figure S2 — DNMT3L and Alcoholism KEGG pathway related protein interaction network in String database. [file Image_2.tif]
